# Mild and severe salt stress responses are age-dependently regulated by abscisic acid in tomato

**DOI:** 10.3389/fpls.2022.982622

**Published:** 2022-10-07

**Authors:** Kristof Holsteens, Isabel De Jaegere, Arne Wynants, Els L. J. Prinsen, Bram Van de Poel

**Affiliations:** ^1^ Division of Crop Biotechnics, Department of Biosystems, University of Leuven, Leuven, Belgium; ^2^ Department of Biology, University of Antwerp, Antwerpen, Belgium; ^3^ KU Leuven Plant Institute, (LPI), University of Leuven, Leuven, Belgium

**Keywords:** salinity, tomato, abscisic acid, ontogeny, abiotic stress, NCED

## Abstract

Salt stress hampers plant growth and development through both osmotic and ionic imbalances. One of the key players in modulating physiological responses towards salinity is the plant hormone abscisic acid (ABA). How plants cope with salinity largely depends on the magnitude of the soil salt content (stress severity), but also on age-related developmental processes (ontogeny). Here we studied how ABA directs salt stress responses in tomato plants for both mild and severe salt stress in leaves of different ages. We used the ABA-deficient mutant *notabilis*, which contains a null-mutation in the gene of a rate-limiting ABA biosynthesis enzyme 9-cis-epoxycarotenoid dioxygenase (NCED1), leading to impaired stomatal closure. We showed that both old and young leaves of *notabilis* plants keep a steady-state transpiration and photosynthesis rate during salt stress, probably due to their dysfunctional stomatal closure. At the whole plant level, transpiration declined similar to the wild-type, impacting final growth. *Notabilis* leaves were able to produce osmolytes and accumulate ions in a similar way as wild-type plants, but accumulated more proline, indicating that osmotic responses were not impaired by the *NCED1* mutation. Besides *NCED1*, also *NCED2* and *NCED6* are strongly upregulated under salt stress, which could explain why the *notabilis* mutant did not show a lower ABA content upon salt stress, except in young leaves. This might be indicative of a salt-mediated feedback mechanism on *NCED2/6* in *notabilis* and might explain why *notabilis* plants seem to perform better under salt stress compared to wild-type plants with respect to biomass and water content accumulation.

## Introduction

The sessile nature of plants forces them to cope with a constantly changing and often hostile environment that hampers growth and development. Salt stress is one of the most important and rapidly emerging plant stressors worldwide (Munns and Tester, 2008; Jamil et al., 2011; Zhu, 2016). Most salt-affected soils have an excess of sodium chloride and provoke a complex biphasic stress response in plants, characterized by altered osmotic and ion balances (Farooq et al., 2017). Despite inherent coping mechanisms, high salinity levels are detrimental to plant growth, development, and thus agricultural productivity (Rengasamy, 2006; Munns and Tester, 2008).

Salt stress evokes a complex interplay between an initial osmotic, and after prolonged exposure, a secondary ion toxicity stress in plants (van Zelm et al., 2020). Although this biphasic response implies temporal separation of sensing fast osmotic changes and subsequent sodium cytotoxicity, actual sodium sensing can occur before the onset of cytotoxicity as indicated by rapid sodium-specific changes in root growth directionality, c.f. halotropism (Munns and Tester, 2008; Galvan-Ampudia et al., 2013). Recent advances indicate that salt stress responses can be signaled by osmotic- and sodium sensors *via* the hyperosmolality-gated Ca^2+^ permeable channel (OSCA1) and the monocation-induced Ca^2+^ increases 1 (MOCA1), respectively (Yuan et al., 2014; Jiang et al., 2019; Zhang et al., 2020). Furthermore, prolonged sodium exposure can induce destabilization of cell wall pectin crosslinking, which can be perceived by the receptor-like kinase (RLK) FERONIA (FER) (Dinneny et al., 2008; Voxeur and Höfte, 2016; Feng et al., 2018).

Osmotic and sodium sensing implies a cytosolic influx of Ca^2+^and subsequent salt stress-specific Ca^2+^ signature, which acts as a secondary messenger to trigger downstream salt stress signaling cascades (Jiang et al., 2019; Zhang et al., 2020). Together with reactive oxygen species (ROS), which accumulate under salt stress, a continuous symplastic progression of Ca^2+^ and ROS waves propagate stress signals to provoke long-distance root-to-shoot and leaf-to-leaf communication (Dat et al., 2000; Choudhury et al., 2017; Devireddy et al., 2018; Wu et al., 2020; Zandalinas et al., 2020). These signals ultimately activate a variety of physiological responses to cope with and survive salt stress (Munns and Tester, 2008; Horie et al., 2012; Park et al., 2016; Yang and Guo, 2018a). Phytohormones, such as abscisic acid (ABA), ethylene, jasmonic acid (JA) and salicylic acid (SA) play a crucial role in coordinating these responses (Verma et al., 2016; Yu et al., 2020). ABA is believed to be a key regulator during salt stress, functioning as a central hub that links environmental cues with endogenous developmental processes (Yu et al., 2020).

Under normal conditions, ABA is maintained at a low level to ensure optimal plant growth and development (Finkelstein, 2013; Yoshida et al., 2015). However, under salt stress, endogenous ABA levels increase rapidly as a consequence of calcium- and ROS-triggered transcriptional up- and downregulation of ABA biosynthesis and catabolism genes respectively (Mulholland et al., 2003; Xiong and Zhu, 2003; Barrero et al., 2006; Golldack et al., 2014; Ismail et al., 2014; Ruiz-Sola et al., 2014; Chen et al., 2020). Moreover, ABA immobilization by protonation in chloride-induced acidification of the cytoplasm can lead to ABA accumulation during NaCl induced salt stress (Geilfus et al., 2015). Nevertheless, upregulation of the rate-limiting ABA biosynthesis gene 9-cis-epoxycarotenoid dioxygenase (*NCED*) plays a crucial role in the fast accumulation of ABA in roots and leaves upon osmotic stress sensing (Xiong and Zhu, 2003). Through ABA-signaling, specific ABA RESPONSIVE ELEMENT-BINDING FACTOR (ABF) transcription factors and additional salt-stress transcription factors (e.g. MYB-, MYC-, NAC-, AP2/ERF-, HD-Zip- and WRKY-type families) control downstream stress-responsive genes (Lan Thi Hoang et al., 2017).

During salt stress, stomatal closure, osmolyte production and ion-transport are three prime physiological plant responses, which could function under control of ABA. ABA modulates ion fluxes of guard cells by Ca^2+^-dependent and independent pathways leading to a decrease in turgor pressure and subsequent stomatal closure to prevent water loss by transpiration (Chen et al., 2020; Bharath et al., 2021). Moreover, the accumulation of osmolytes, such as proline and glycine betaine, aid plants in coping with salt stress by increasing the cellular osmotic pressure and maintaining turgor (Sharma et al., 2019). ABA plays a significant role in regulating proline biosynthesis by modulating pyrroline-5-carboxylate synthase (*P5CS*) and P5C reductase (*P5CR*) (Sripinyowanich et al., 2013; Pál et al., 2018). Furthermore, ABA also plays a role in the biosynthesis of other osmolytes such as polyamines and sugars (Thalmann et al., 2016; Golestan Hashemi et al., 2018). During salt stress, cytotoxic sodium levels hamper ion homeostasis, leading to nutrient deficiencies, in particular K^+^ deficiency, and subsequent deterioration of plant growth (Wang et al., 2013). ABA modulates Ca^2+^ influx at the plasma membrane, which induces the salt overly sensitive (SOS) pathway, leading to sodium export from roots (Edel and Kudla, 2016). Moreover, ABA stimulates the deposition of suberin at the endodermis to hamper apoplastic Na^+^ migration towards the stele (Barberon et al., 2016; Doblas et al., 2017). Finally, ABA promotes cytosolic K^+^ influx by activating potassium import channels KAT1 and AKT1 (Osakabe et al., 2014; Yang and Guo, 2018b).

The success of salt stress coping mechanisms in retaining plant growth and survival vastly depends on stress severity and plant or leaf age (ontogeny). Most salt-affected soils are only mildly saline (Omuto et al., 2020), which does not evoke visible salinity symptoms, yet leads to yield losses (Machado and Serralheiro, 2017; Zörb et al., 2019). Furthermore, age-related elements can mask unknown developmentally controlled adaptation responses towards abiotic stress (Rankenberg et al., 2021). Although these under-explored factors (mild salinity and ontogeny) are important to understand to grasp the holistic coping mechanisms of plants towards salt stress they are often neglected in research (Mulholland et al., 2003; Amjad et al., 2014; Raziq et al., 2022). How ABA relates to stress severity and whether it functions in an age-dependent way under salt stress is studied here.

## Materials and methods

### Plant material and growth conditions

Seeds of tomato cultivar Ailsa craig (*Solanum lycopersicum*) and *notabilis*, harboring a null mutation in the *NCED1* gene in the Ailsa craig background, were germinated in soil and after 14 days transferred to Rockwool blocks. Plants were grown in a controlled greenhouse with relative humidity set to be 70% and 65% during the day (6 am – 10 pm) and night respectively, and a minimum temperature of 17°C and ventilated once exceeded 18°C. When irradiation levels were below 250 W/m^2^, additional light was provided with SON-T lamps (Phillips). Plant nutrition was given by supplementing a fertigation solution with an electrical conductivity (EC) of 2.5 dS/m and a pH of 5.75. The nutrient solution was composed of K^+^ 9.1 mM, Ca^2+^ 6.5 mM, Mg^2+^ 1.95 mM, $NO_{3}^{-}$ 16.25 mM, $PO_{4}^{3-}$ 1.95 mM, $SO_{4}^{2-}$ 3.9 mM, $MoO_{4}^{2-}$ 0.1 µM, Zn^2+^ 8.6 µM, Cu^2+^ 2.6 µM, Mn^2+^ 139.8 µM, $BO_{3}^{3-}$ 54.4 µM, Fe^2+^ 79.9 µM. Fertigation frequency was programmed as follows: (1) every 45 minutes between 7 and 10 am, or 30 minutes when radiation exceeds 250 W/m2; (2) every 60 minutes between 10 am and 1 pm, or 45 minutes when radiation exceeds 250 W/m2.; (3) every 60 minutes between 1 and 5 pm, or 45 minutes when the radiation sum exceeds 150 J/cm2.

### Salt treatment and real-time EC measurements

Tomato plants at the eight-leaf stage were divided into three treatment groups: control, mild and severe salt stress. Each group contained 10 plants (n = 10) and was randomized in the greenhouse to minimize position and micro-climate effects. The control solution (EC 2.5 dS/m) was supplemented with a concentrated NaCl stock solution (6.16 M) to reach mild salinity (EC of 5 dS/m; [NaCl] = 47 mM) and sever salinity (10 dS/m; [NaCl] = 94 mM). The treatments started at 10 AM with 5-minutes of continuous drip irrigation to ensure the Rockwool blocks were drained and saturated with salt solution. Subsequently, the salt treatment was applied for 7 days by providing automated drip irrigation as described above.

The EC of the Rockwool block of one plant per treatment was monitored with an EC sensor (GS3 and Terros12, Meter). These sensors were first calibrated by saturating Rockwool blocks with known fertigation solutions with an EC ranging from 0 dS/m – 15 dS/m in steps of 2.5 dS/m. Rockwool blocks were air-dried on lysimeters to obtain real-time volumetric water content (VWC) measurements. When dried, the relationship between real-time VWC and known EC and raw sensor VWC and EC was calculated using the bulk and pore water EC relationship formulas as indicated by the work of (Hilhorst, 2000), specifically for Rockwool substrate.

### Genotyping

DNA was extracted from 50 mg snap-frozen pulverized fresh leaf material using the E.Z.N.A.^®^ SP Plant DNA Kit (Omega Biotek). Next, the quality and concentration of the extracted DNA were assessed using the Nanodrop 2000 (Thermo Fisher Scientific). Afterward, a polymerase chain reaction (PCR) was performed to amplify a 500 bp amplicon, flanking the NCED1 mutation, using DreamTaq Green PCR Master Mix (2X) (Thermo Scientific) and specific forward (CTTGAACACCCTTTGCCGAA) and reverse (GAAACTGGGTCGAGCTTTGG) primers (IDT). Samples with a verified amplicon length, using gel electrophoresis, were gel-extracted and sequenced (LGC genomics).

### Protein sequence alignment and phylogenetic analysis

Candidate NCED protein sequences were retrieved by blast using the NCED1 (Solyc07g056570) as a query on the SolGenomics website, using the tomato genome (release ITAG 4.0), and arabidopsis.org website, using the Arabidopsis genome (release Araport 11). Retrieved CCD and NCED protein sequences of tomato and Arabidopsis were aligned in Geneious (v10.2.2) using the MUSCLE (v3.8.425) tool. The phylogenetic tree was built in Geneious using the Jukes-Cantor genetic distance model and bootstrapping (1000 replicates).

### Plant biometry and real-time plant transpiration

For each plant (n = 10 biological replicates) per treatment (control, mild and severe salinity), stem growth, and above-ground plant fresh and dry weight were recorded. Whole plant transpiration of individual plants was inferred from real-time lysimeters, consisting of 30 custom-made scales with a logging frequency of 30 s. These scales are composed of a 5 kg load cell controlled by a microcontroller (Arduino Uno). The scale housing contains a drain to remove excess irrigation solution, and Rockwool blocks were encapsulated with aluminum foil to prevent evaporation. Raw transpiration data was analyzed using an R-script that removes irrigation points, calculates, and models weight loss over time, and generates a transpiration curve based on the first derivative of the latter. Daily transpiration rates were calculated by integration of the area underneath the diurnal transpiration rate curves.

### Leaf biometry, transpiration and CO_2_ gas exchange

To incorporate ontogenetic responses, we analyzed leaf numbers 3 and 8, starting to count from the oldest leaf (bottom) upwards, omitting the two cotyledons, for each plant (n = 10 biological replicates per treatment). These leaf ages represent old (no. 3) and young leaves (no. 8). Individual leaf fresh and dry weight was measured at the end of the experiment. Leaf area was quantified at the end of the experiment (day 7) through analysis of top view images (Nikon D3200) using ImageJ. Leaf photosynthesis and transpiration rate were determined for individual leaflet patches with an LCi compact portable photosynthesis system (ADC Bioscientific).

### Leaf osmolality

The total osmolality of old and young leaves (n = 10 biological replicates) per treatment was quantified by taking the supernatant of snap-frozen and pulverized fresh leaf samples after centrifugation at 17.000 x g for 20 min. The osmolality of 50 µL leaf sap was measured using a freezing point osmometer (Osmomat 3000, Gonotec).

### Leaf proline content

Proline content of old and young leaves (n = 10 biological replicates) per treatment was quantified using a spectrophotometric method based on the reaction with ninhydrin (2,2-dihydroxyindane-1,3-dione) in an acidic medium, as described by Ábrahám et al. (2010), with slight modifications. First, proline was extracted from 50 mg of snap-frozen pulverized fresh leaf material using 25 µL pure ethanol. After homogenization by vortexing, the samples were incubated at 65°C on a shaking heat block for 20 min and centrifuged for 5 min at 17.000 x g. Next, the coloring reaction was done by adding 50 µL of the supernatant to 100 µL of the freshly made reaction mix, comprised of 1% (w/v) ninhydrin dissolved in 60% (v/v) acetic acid and 20% (v/v) ethanol. After homogenization, the samples were placed in a shaking heat block at 95°C for 20 minutes, covered with aluminum foil to avoid ninhydrin degradation by light. The reaction was immediately stopped on ice and centrifuged for 1 min at 2500 rpm at 4°C. Finally, 100 μL of supernatant was transferred to a 96-well microtiter plate and absorbance was measured at 509 nm using a plate spectrophotometer (Spectramax 384 plus, Molecular Devices).

### Leaf ion content

For ion analysis, 125 mg of dried and ground leaf powder (n = 10 biological replicates per treatment) was transferred to a crucible and subsequently incinerated in a muffle furnace (MR 170 E, Heraeus) at 500°C for 4 h. After cooling down, the ash was analytically transferred and dissolved in 50 mL of 2M HCl (37%, VWR Chemicals) and placed in a water bad at 50°C for 2 h. The samples were diluted 50 times for the determination of Na^+^ and K^+^, and 8 times for the determination of Ca^2+^. The ion content was quantified on a SOLAAR 969 atomic absorption spectrometer (Unicam) using a photomultiplier tube (PMT) and the following specifications; K^+^; Na^+^ and K^+^ hollow-cathode lamp (HCL) with a max current of 8 mA (100% during measurement) using a wavelength of 766.5 nm, a bandwidth of 0.5 nm and an air/acetylene fuel flow of 0.8 L/min. Na^+^; Na^+^ and K^+^ hollow-cathode lamp (HCL) with a max current of 8 mA (75% during measurement) using a wavelength of 589 nm, a bandwidth of 0.2 nm and an air/acetylene fuel flow of 0.8 L/min. Ca^2+^; Ca^2+^ and Mg^2+^ hollow-cathode lamp (HCL) with a max current of 6 mA (100% during measurement) using a wavelength of 422.7 nm, a bandwidth of 0.2 nm and an air/acetylene fuel flow of 0.8 L/min. Additionally, the calcium measurements were performed in the presence of 2% 0.36 M lanthanum(III)oxide to eliminate phosphate interference.

### Leaf ABA content

Leaf ABA content was measured (n = 5 biological replicates per treatment) using 50 mg of snap-frozen pulverized fresh leaf material. d-6 ABA (150 pmol, 3′,5′,5,7′,7′,7′-d-6-ABA, National Research Council Canada, Saskatoon, Saskatchewan, Canada) was added as an internal standard. After overnight extraction in 5 ml 80% MeOH, samples were centrifuged (20 min, 15,000 g, 4°C, in an Eppendorf 5810R centrifuge, Eppendorf, Hamburg, Germany), the supernatants was acidified using 5 mL of 6.0% v/v formic acid and loaded on a reversed-phase (RP) C18 cartridge (500 mg, BondElut Varian, Middelburg, The Netherlands). ABA was eluted with 5 mL of diethyl ether and dried under a nitrogen stream (TurboVap LV Evaporator, Zymark, New Boston, MA, USA). Samples were derivatised using N-(3-Dimethylaminopropyl)-N′-ethylcarbodiimide (EDC, Sigma, 0.1 mg/sample, pH 7, 1 h, 37°C under continuous shaking, Eppendorf thermomixer) to improve analysis sensitivity and analyzed using an Acquity UPLC system linked to a TQD triple quadrupole detector (Waters, Milford, MA, USA) equipped with an electrospray interface in positive ion mode. Samples (6.0 μL) were injected on an Acquity UPLC BEH C18 RP column (1.7 μm, 2.1 × 50 mm, Waters) using a column temperature of 40°C and eluted at 0.42 mL with the following gradient of 0.1% FA/H2O (solvent A) and 0.1% FA/ACN (solvent B): 0−0.8 min isocratic 92/8 A/B; 0.8−5 min linear gradient to 60/40 A/B; 5-5.5 linear gradient to 10/90 A/B. Quantitative analysis was obtained by multiple reactant monitoring of selected transitions, based on the MH+ ion (dwell time 0.02 s) and the most appropriate compound-specific product ions (420>349 and 420>304 for ABA-EDAC and 426>355 and 426>310 for d6-ABA-EDAC). All data were processed using Masslynx/Quanlynx software V4.2 (Waters). Data are expressed in picomoles per gram fresh weight (pmol g^-1^ FW).

### RT-qPCR gene expression analysis

For *NCED1*, *NCED2* and *NCED6* quantitative gene expression analysis, mRNA was extracted from 50 mg snap-frozen pulverized fresh leaf material (n = 5 biological replicates per treatment) using the Genejet Plant RNA Purification Mini Kit (Thermo Fisher Scientific) and subsequent DNA removal using the RapidOut DNA Removal Kit (Thermo Fisher Scientific). The quality and concentration of the extracted RNA were measured using the Nanodrop 2000 (Thermo Fisher Scientific). Next, mRNA was converted to copy DNA (cDNA) using the iScript cDNA Synthesis Kit (BioRad). Finally, expression levels were quantified by real-time PCR using the SsoAdvanced Universal SYBR Green Supermix (BioRad), specific primers (IDT, **Supplementary Table 1**) on a CFX96 real-time PCR detection system (BioRad). Gene expression was normalized against the average expression of two reference genes (*ACT* and *EGFR*) and quantified based on a calibration curve developed by a cDNA mix sample (of all samples) in 3 orders of magnitude dilution.

### Data analysis and statistics

Data analysis was performed using Graphpad (Version 9, Delta Prism). Test for normal distributed data and equal variance between the groups was performed using the Shapiro-Wilk test and Spearman’s test for heteroscedacity, respectively. If the data passed, a two-way ANOVA and subsequent Sídák’s multiple comparison test was used to test significance (p < 0.05). Nonparametric data was analysed using the Mann-Whitney test for significance (p <0.05).

## Results

### Characterization of the tomato *NCED* gene family and *notabilis* mutation

**Figure 1:**
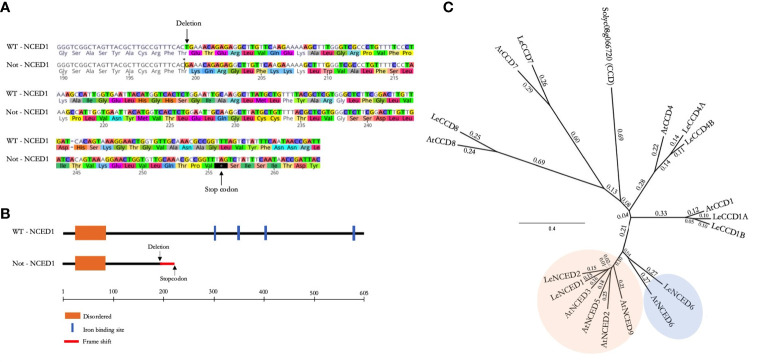
The NCED gene family and *notabilis* genotyping. **(A)** Protein sequence alignment of tomato NCED1 wild-type and *notabilis*, showing a single amino acid deletion (Thr199) and a premature stop codon. Arrows mark the deletion and early stop codon. **(B)** Schematic representation of the wild-type and *notabilis* (truncated) NCED1 protein, including functional domains. **(C)** Phylogenetic tree of the CCD and NCED proteins of tomato and Arabidopsis. The two NCED clusters are shown in orange and blue.

Before assessing ABA-related ontogenic responses towards mild and severe salinity, we first genotyped the *notabilis* mutant. The null mutation, generated by x-ray mutagenesis, in the *notabilis NCED1* gene is reported to consist of a single base-pare deletion at T596 in the Thr199 codon (Burbidge et al., 1999). We confirmed *notabilis* contained this T deletion, resulting in a frameshift that leads to an early stop codon at position 256 (**Figure 1A**). This premature stop codon causes a truncated protein that loses the enzyme’s iron-binding sites (**Figure 1B**). Phylogenetic analysis revealed that the tomato genome harbors three putative *NCED* genes compared to five members retrieved in the Arabidopsis genome (**Figure 1C**). The NCED protein family clusters in 2 clades with LeNCED1 (Solyc07g056570) and LeNCED2 (Solyc08g016720) belonging to clade 1 where they closely relate to AtNCED3 (AT3G14440) with respectively 71.2 and 70.0% sequence similarity. LeNCED6 (Solyc05g053530; Sussmilch and McAdam, 2017) belongs to clade 2 and shows 57.4% sequence similarity with AtNCED6 (AT3G24220) (**Figure 1C**).

### *Notabilis* and wild-type plants have similar plant growth responses during salt stress

**Figure 2:**
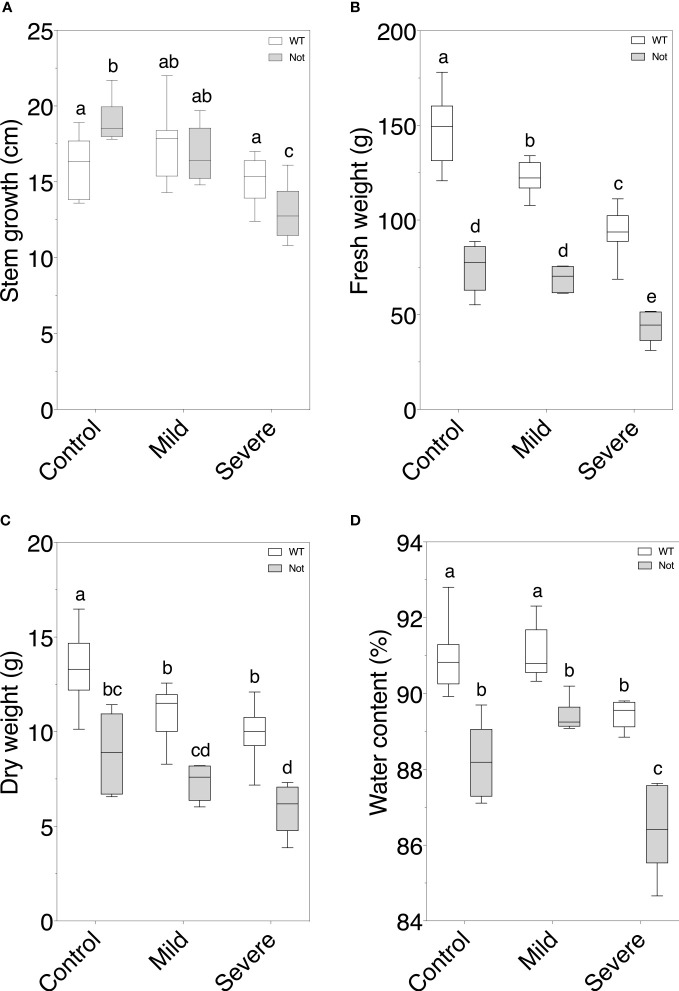
Assessment of plant growth parameters in wild-type and *notabilis* plants during salt stress. **(A)** Stem length increase over the course of 7 days (n = 10), **(B)** fresh weight (n = 10), **(C)** dry weight (n = 10) and **(D)** water content (n = 10) after 7 days under control (EC of 2.5 dS/m), mild (EC of 5 dS/m; [NaCl] = 47 mM) and severe (EC of 10 dS/m; [NaCl] = 94 mM) salt stress conditions for the wild-type (white) and *notabilis* (gray). Significant differences are indicated with different letters (α = 0.05).

After confirming the *notabilis* genotype, we analyzed how the hampered *nced1* protein would impact plant growth under mild and severe salt stress. Our experimental setup provided the desired experimental conditions, i.e control (EC of 2.5 dS/m), mild (EC of 5 dS/m) and severe (EC of 10 dS/m), to the Rockwool blocks and stayed stable after 7 days of continuous salt stress (**Supplementary Figure 1**). Despite the *NCED1* null mutation in *notabilis*, minor differences in growth responses towards salt stress were observed compared to wild-type plants (**Figure 2**). Although wild-type plants showed no decrease in stem growth under both mild and severe salinity, *notabilis* did show a significant decrease under severe salinity (**Figure 2A**). In general, besides the lower fresh weights of *notabilis* plants compared to the wild-type plants, they showed a similar decrease in fresh weight under salt stress as the wild-type (**Figure 2B**). A similar pattern was observed for dry weight loss (**Figure 2C**). Interestingly, the plant water percentage was only significantly affected by severe salt stress for both wild-type and *notabilis* plants (**Figure 2D**).

### Salt stress impacts leaf growth in an age-dependent way in *notabilis*

**Figure 3:**
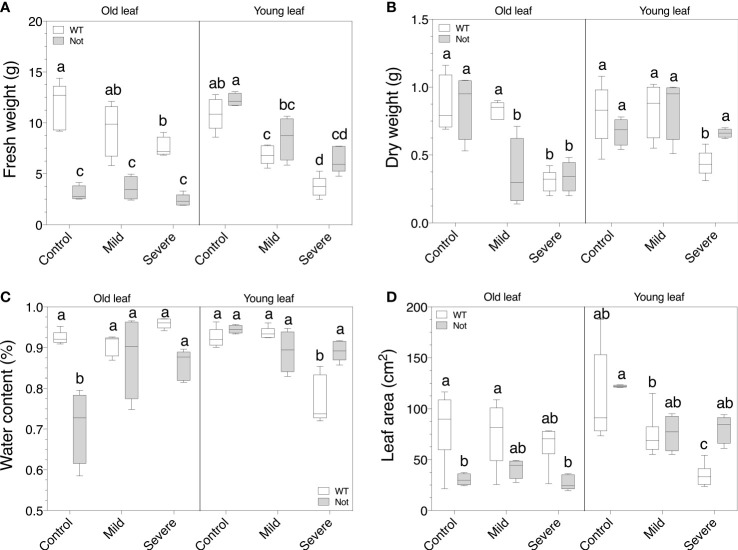
Assessment of leaf growth parameters for wild-type and notabilis plants under salt stress. **(A)** Leaf fresh weight (n = 10), **(B)** leaf dry weight (n = 10), **(C)** leaf water content (n = 10) and **(D)** leaf area (n = 10) of old (leaf no. 3) and young (leaf no. 8) leaves after 7 days under control (EC of 2.5 dS/m), mild (EC of 5 dS/m; [NaCl] = 47 mM) and severe (EC of 10 dS/m; [NaCl] = 94 mM) salt stress conditions for the wild-type (white) and *notabilis* (gray). Significant differences are indicated with different letters (α = 0.05).

Despite the comparable whole-plant growth responses of the *notabilis* mutant under salt stress, we evaluated leaf-specific growth responses for old (leaf nr 3) and young (leaf nr 8) leaves to assess whether there are otogenic salt stress responses. Old leaves of *notabilis* plants have a lower fresh weight compared to wild-type plants under control conditions, which is not the case for young leaves (**Figure 3A**). Salinity drastically reduces leaf fresh weight, for both old and young leaves in wild-type plants (**Figure 3A**). This salt effect on fresh weight was only observed in young *notabilis* leaves (**Figure 3A**). Oppositely, the dry weight of old *notabilis* leaves dropped during salt stress, similar as the wild-type, while young *notabilis* leaves showed no drastic decline (**Figure 3B**). Interestingly, *notabilis* showed an increased water content under mild and severe salinity in old leaves compared to the wild-type, due to the loss of dry matter (**Figure 3C**). Severe salt stress led to a drop in leaf water content of young wild-type leaves, suggesting that young leaves lost more water than dry matter (**Figure 3C**). In contradiction, *notabilis* leaves did not show a decline in leaf water content, despite old leaves already having a lower water content in unstressed conditions (**Figure 3C**). Water content stayed stable for young leaves and even increased for old leaves during mild and severe salt stress, suggesting that salt stress is able to evoke a water-saving mechanism in *notabilis* leaves (**Figure 3C**). Moreover, similar as the dry weight and water content, the leaf area of young *notabilis* leaves did not drop under salt stress compared to the wild-type, indicative of a growth-saving feature of young *notabilis* leaves (**Figure 3D**).

### *Notabilis* maintains high leaf transpiration but has a reduced whole-plant transpiration during salt stress

**Figure 4:**
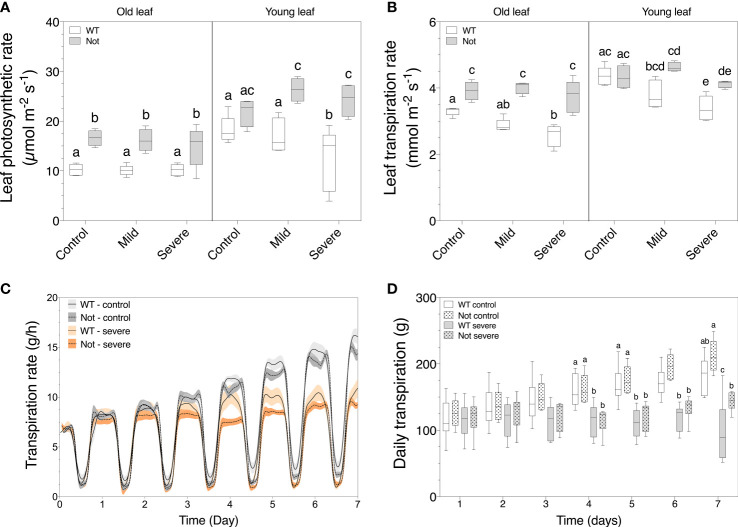
Plant transpiration and leaf photosynthetic and transpiration rate of old (leaf no. 3) and young (leaf no. 8) leaves in wild-type (white) and *notabilis* (gray) after 7 days under control (EC of 2.5 dS/m), mild (EC of 5 dS/m; [NaCl] = 47 mM) and severe (1EC of 10 dS/m; [NaCl] = 94 mM) salt stress conditions. **(A)** Leaf photosynthetic rate (n = 10), **(B)** leaf transpiration rate (n = 10), **(C)** real-time plant transpiration rate (n = 10) and **(D)** daily transpiration rate (n = 10) during 7 days of salt stress. Significant differences are indicated with different letters (α = 0.05). Lines in **(C)** represent average leaf angles +/- the confidence interval (90%).

To further investigate the different water balances in *notabilis* plants and leaves, we compared leaf and whole plant physiology. Salt stress reduced wild-type leaf photosynthesis only in young leaves (**Figure 4A**) and led to a drop in transpiration in both old and young wild-type leaves (**Figure 4B**). Interestingly, salt stress did not influence leaf photosynthesis and transpiration in *notabilis* leaves (**Figures 4A, B**). This can be explained by the hampered stomatal closure in the *notabilis* mutant that allows leaves to maintain high rates of gas exchange (Shi et al., 2015). Despite the unaffected leaf transpiration rates, whole plant transpiration was equally reduced for *notabilis* and wild-type plants under severe salinity starting from day 4 onwards (**Figures 4C, D**). Moreover, when calculating the daily transpiration rates, *notabilis* and wild-type plants both showed a similar decline in plant transpiration (**Figure 4D**).

### *Notabilis* is able to maintain leaf osmotic regulation under salt stress

**Figure 5:**
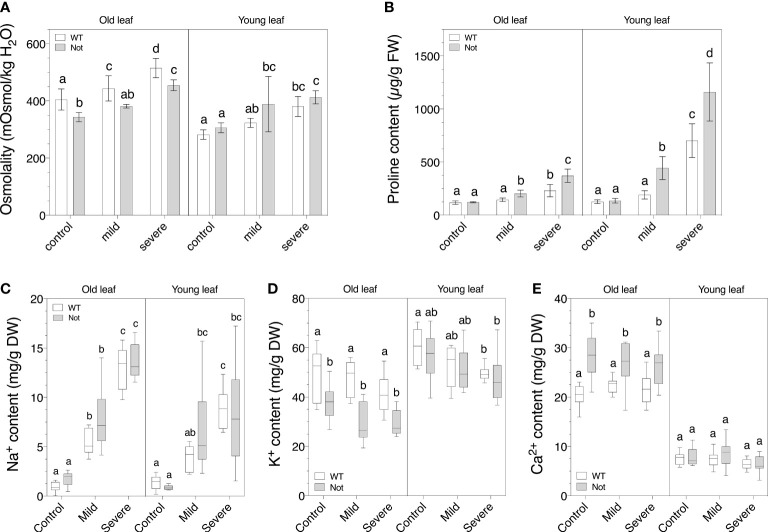
Osmotic balance of old (leaf no. 3) and young (leaf no. 8) leaves in wild-type (white) and *notabilis* (gray) after 7 days under control (EC of 2.5 dS/m), mild (EC of 5 dS/m; [NaCl] = 47 mM) and severe (EC of 10 dS/m; [NaCl] = 94 mM) salt stress conditions. **(A)** Leaf osmolality as a measurement for total osmolyte content (n = 10), **(B)** leaf proline content (n = 10), **(C)** leaf sodium content (n = 10), **(D)** leaf potassium content (n = 10) and **(E)** leaf calcium content (n = 10). Significant differences are indicated with different letters (α = 0.05).

To further address the disrupted leaf photosynthetic and transpiration rate of *notabilis* mutants compared to wild-type plants, we analyzed the osmotic changes in leaves of different ages during mild and severe salt stress. The total osmolyte content in young and old leaves increased comparably between wild-type and *notabilis* leaves under salt stress (**Figure 5A**). Despite the lack of osmotic differences between *notabilis* and wild-type leaves, proline accumulated more in *notabilis* leaves under both mild and severe salt stress, especially in young leaves (**Figure 5B**). This suggests that *notabilis* might preferably use proline to control its osmotic potential.

Previously it was reported that ion transport is controlled by ABA to mediate ion toxicity during salt stress (Yu et al., 2020). Our results show that there was no large difference in ion content between *notabilis* and wild-type leaves for Na^+^, K^+^ and Ca^2+^ under salt stress (**Figures 5C–E**). In general, Na^+^ levels increased when salt stress became more severe, while K^+^ levels dropped slightly and Ca^2+^ levels remained unchanged. Noteworthy, under control conditions, old *notabilis* leaves had a lower K^+^ content and a higher Ca^2+^ content compared to wild-type leaves, and this difference was not present in young leaves (**Figures 5D, E**). We can conclude that that *notabilis* plants have a slightly different age-related regulation of their ion homeostasis but seem to have a similar ion coping mechanism during salt stress.

### Impaired NCED1 is not sufficient to affect salt stress-induced ABA accumulation

**Figure 6:**
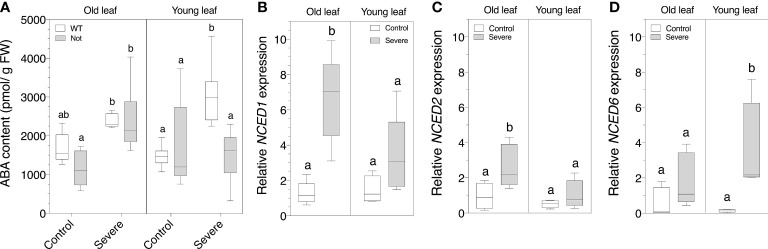
Quantification of leaf ABA content and *NCED* transcript levels of old (leaf no. 3) and young (leaf no. 8) leaves in wild-type (white) and notabilis (gray) after 7 days under control (EC of 2.5 dS/m) and severe (EC of 10 dS/m; [NaCl] = 94 mM) salt stress conditions. **(A)** Whole leaf ABA content (n = 5). **(B-D)** Relative expression in the wild-type plants of **(B)** *NCED1* (n = 5), **(C)** *NCED2* (n = 5) and **(D)** *NCED6* (n = 5) under control (white) and severe (gray) salt stress conditions. Significant differences are indicated with different letters (α = 0.05).

Although we observed age-related physiological and biochemical differences between *notabilis* and wild-type plants (**Figures 2**-**5**), *notabilis* plants are still able to respond to salt stress, despite a non-functional NCED1. An ontogenic ABA analysis showed that *notabilis* plants are able to produce ABA, similar to wild-type plants, and that old *notabilis* leaves are able to accumulate ABA under severe salt stress in the same way as old wild-type leaves (**Figure 6A**). Young *notabilis* leaves are not able to accumulate higher levels of ABA during severe salt stress (**Figure 6A**). These observations suggest that old *notabilis* leaves have a mechanism to increase their ABA levels, independent of NCED1. To investigate the role of the other NCED homologs, we performed a gene expression analysis of the three *NCED* genes of tomato (**Figure 1**). We found that *NCED1* and *NCED2* are upregulated in old wild-type leaves during salinity (**Figures 6B, C**). This might suggest that NCED2 could contribute to the higher ABA levels in old *notabilis* leaves (**Figure 6A**). Young leaves, on the other hand, show an upregulated *NCED6* expression upon severe salinity (**Figure 6D**), indicating that the *NCED* gene family is ontogenetically regulated during salt stress.

## Discussion

Soil salinization induces a wide array of detrimental effects on plant growth and development for which plants have acquired numerous coping mechanisms to counteract and survive the harmful effects of both osmotic and ion toxicity stress during salinity (Parida and Das, 2005; Munns and Tester, 2008). However, the success of these coping mechanisms vastly depends on stress severity and plant or leaf age (ontogeny), c.f. age-related developmental elements can mask certain salt adaptation responses (Machado and Serralheiro, 2017; Zörb, Geilfus and Dietz, 2019; Omuto et al., 2020; Rankenberg et al., 2021). One of the key regulators during salt stress is ABA, but to date its involvement in the ontogenic regulation of salt stress responses is still unclear (Yu et al., 2020).

### The tomato NCED family directs leaf age-dependent ABA accumulation under salt stress

To address the involvement of ABA in age-related salt stress coping mechanisms of tomato, we used the *notabilis* mutant, harboring a null mutation in the rate-limiting ABA biosynthesis gene *NCED1* (Burbidge et al., 1999). The truncated NCED1 resulted in a lower ABA accumulation only in young *notabilis* leaves during severe salt stress (**Figure 6**), suggesting that impairment of NCED1 is not sufficient to prevent ABA production in older leaves. Previously, ABA accumulation under drought (Neill and Horgan, 1985) and salt (Mulholland et al., 2003) stress was also observed in *notabilis* plants. We now revealed that this stress-induced ABA accumulation is leaf-age specific in *notabilis*. The accumulation of ABA in old leaves should originate from local ABA production or ABA translocation from e.g. the roots. However, recent findings largely ruled out long-distance root-to-shoot ABA transport during salt or osmotic stress (Waadt et al., 2014), indicating that local ABA production in the leaves drives stress responses (McAdam et al., 2016). It was shown that the small peptide CLAVATA3/ESR-RELATED 25 (CLE25) might function as a long-distance root-to-shoot signal that induces ABA biosynthesis using the BARELY ANY MERISTEM (BAM) receptor-kinases (Takahashi et al., 2018).

Although earlier findings indicated the presence of two additional NCED homologs in tomato (Burbidge et al., 1999), their classification has been conflicting (Son et al., 2016). Our phylogenetic analysis of the NCED family showed the presence of two other NCED homologs in tomato (**Figure 1**), corroborating findings of a recently published bioinformatics study of the larger carotenoid cleavage oxygenase (CCO) superfamily (Yao et al., 2022) and in agreement with Priya and Siva (2015) and Sussmilch and McAdam (2017). In *Arabidopsis thaliana*, the NCED family contains 5 members (Tan et al., 2003; Ali et al., 2020) and research indicated that At*NCED3* is upregulated under salinity stress, *via* ABA-dependent and ABA-independent signals (Barrero et al., 2006). We showed that under salt stress the closely related *NCED1* and *NCED2* are mostly upregulated in old leaves, whereas *NCED6* is mostly upregulated in young leaves (**Figure 6**). Our results indicate that the lack of a functional NCED1 in *notabilis* might induce a feed-back mechanism on NCED2 and NCED6 which can activate ABA biosynthesis during salt stress, albeit leaf-age specific. Although most studies in *Arabidopsis thaliana* investigate the role of AtNCED6 in seed dormancy (Lefebvre et al., 2006; Chauffour et al., 2019; Yang et al., 2022), Le*NCED6* upregulation has been shown in tomato leaves during heat stress (Chi et al., 2021). However, to what extent NCED2 secures ABA biosynthesis under salt stress and why the upregulation of *NCED6* under salt stress did not promote ABA accumulation in young *notabilis* leaves remains elusive. An earlier study by Mulholland et al. (2003) also examined the relationship between ABA and salt stress by using the ABA-deficient tomato mutant *notabilis*. Their findings indicate that although ABA levels are seemingly less affected by the dysfunctional NCED1 in roots, ABA levels in leaves, although lower than wild-type, still accumulate during salt stress. Our results not only confirm these findings in leaves, but also show that there is an ontogenic relationship towards ABA accumulation during salt stress. Moreover, together with their findings, a spatial expression pattern between roots and leaves might suggest that NCEDs operates differentially in roots and leaves in an ontogenic fashion.

### *Notabilis* compensates transpiration losses of older leaves to secure growth

In the absence of stress, *notabilis* plants show clear phenotypic differences compared to wild-type plants, i.e. wilting phenotype with thinner longer stems and epinastic leaves, as a result of the dysfunctional NCED1 protein (Burbidge et al., 1999). The *notabilis* mutation results in an abnormal stomatal behavior (Tal, 1966) and higher leaf transpiration rates, explaining the wilted phenotype (Neill and Horgan, 1985; Burbidge et al., 1999). In contradiction, our real-time lysimeter measurements showed that *notabilis* plants have a similar whole plant transpiration rate as wild-type plants (**Figure 4**). We showed that only old leaves have an impaired stomatal closing and therefore have a higher leaf transpiration rate, while young *notabilis* leaves retain normal transpiration rates (**Figure 4**). The higher transpiration rate of old leaves also leads to a higher photosynthesis rate (**Figure 4**). Previously, Ntatsi et al. (2014) showed that *notabilis* scions grafted on *notabilis* root-stocks (*notabilis* self-graft) also had a higher leaf transpiration and photosynthesis rates compared to wild-type grafts, implying that perhaps a higher photosynthesis rate could benefit growth of *notabilis* plants. However, our results show that *notabilis* plants have a lower fresh and dry weight, and lower water content in contrast to wild-type plants (**Figure 2**), indicating that the extra photosynthetic assimilates are likely used differently. We found that the above-ground loss in plant biomass of *notabilis* is predominantly caused by having smaller older leaves (**Figure 3**), which was also recorded by Mulholland et al. (2003). Additionally, *notabilis* self-grafts showed a higher root biomass compared to the wild type (Ntatsi et al., 2014). Conceivably, *notabilis* plants invests the extra energy obtained from a higher photosynthesis rate of old leaves in the development of sinks, such as new leaves or additional roots, to secure water uptake and compensate excessive transpiration losses.

### *Notabilis* shows leaf age-dependent coping mechanisms during salt stress

Despite the impaired *NCED1* gene in *notabilis*, the effect of salt stress on plant growth and whole-plant transpiration is minor and comparable to wild-type plants. However, our results indicate that *notabilis* plants show a leaf age-dependent response during salt stress. Salinity mainly impacts the fresh weight of young leaves and leads to dry weight loss of old leaves. During salt stress, both old and young leaves of *notabilis* maintain a high rate of leaf transpiration (**Figure 4**), probably due to the inability of the plant to close its stomata. Although this implies a higher rate of water loss, open stomata might explain the high photosynthetic rate in old and young leaves during salt stress. However, despite higher transpiration rates of *notabilis* leaves under salt stress, no difference in whole plant transpiration rate was observed (**Figure 4**). Furthermore, leaf water content stays stable in young leaves and even increased in old leaves during mild and severe salt stress (**Figure 3**), indicating water uptake under salt stress conditions is sustained in *notabilis*. The ability to retain water under a high transpiration load while salt stress limits water uptake is likely not the result of increased osmoregulation in *notabilis* leaves, as no differences in both total osmolyte content and ion content under salt stress were observed (**Figure 5**). However, we observed a significant increase in proline content in both old and young *notabilis* leaves under salt stress compared to wild-type leaves (**Figure 5**), which might help to facilitate osmoregulation in *notabilis* plants. It was shown before that ABA plays a role in the biosynthesis of proline under stress (Sripinyowanich et al., 2013; Pál et al., 2018). The higher proline accumulation in *notabilis* leaves could be explained by the feed-back mechanism of the other *NCED* genes under salt stress to sustain ABA levels. Alternatively, the higher amount of root development in *notabilis* plants (Ntatsi et al., 2014), could aid in securing water during salt stress. Previously, it was shown that *notabilis* self-grafts have a lower sugar, starch and polyamine content under non-stressed conditions (Ntatsi et al., 2014). However, the role of these other osmolites or the involvement of aquaporins in *notabilis* plants facing salt stress remains to be investigated.

## Conclusion

Our results show that tomato plants utilize three *NCED* genes that are stress-specific and age-dependently regulated. *Notabilis* mutants harboring a dysfunctional NCED1 are still able to accumulate ABA under salt stress in an age-dependent way. Furthermore, although plant growth is not hampered by *nced1* during salt stress, we show that there are physiologic differences dependent on leaf age and that they might contribute to altered coping mechanisms and strategies in tomato plants. Leaf transpiration and photosynthesis are sustained in *notabilis* plants facing salinity stress, while leaf ion content was impacted similarly as control plants. *Notabilis* leaves likely use proline as osmoprotectant, independent of NCED1 functionality, to compensate for leaf transpiration losses.

## Data availability statement

The original contributions presented in the study are included in the article/**Supplementary Material**. Further inquiries can be directed to the corresponding author.

## Author contributions

KH, BVdP designed the research; KH, IDJ, AW performed research; EP performed ABA analyses; KH, IDJ, AW, BVdP analyzed the data; KH, BVdP wrote the manuscript with the input of all authors. All authors contributed to the article and approved the submitted version.

## Funding

The research was financially supported by the Interreg Vl-Nl project GROW, the Research Foundation Flanders (grant nr G092419N) and KU Leuven (grant nr C14/18/056).

## Conflict of interest

The authors declare that the research was conducted in the absence of any commercial or financial relationships that could be construed as a potential conflict of interest.

## Publisher’s note

All claims expressed in this article are solely those of the authors and do not necessarily represent those of their affiliated organizations, or those of the publisher, the editors and the reviewers. Any product that may be evaluated in this article, or claim that may be made by its manufacturer, is not guaranteed or endorsed by the publisher.
