# Supplementary material for: Mild and severe salt stress responses are age-dependently regulated by abscisic acid in tomato
Source: Front Plant Sci. 2022 Oct 7;13:982622. doi: 10.3389/fpls.2022.982622 (PMC9585276; doi:10.3389/fpls.2022.982622)
Supplement: Supplementary file 1 [file DataSheet_1.docx]

**Supplementary Material**

**Mild and severe salt stress responses are age-dependently regulated by abscisic acid in tomato**

**Kristof Holsteens^1^, Isabel de Jaegere^1^ , Arne Wynants^1^, Els Prinsen^2^, Bram Van de Poel^1,3^**

^1^ Division of Crop Biotechnics, Department of Biosystems, University of Leuven, Willem de Croylaan 42, 3001 Leuven, Belgium

^2^ Department of Biology, University of Antwerp, Groenenborgerlaan 171, 2020 Antwerpen, Belgium

^3^ KU Leuven Plant Institute, (LPI), KU Leuven, Kasteelpark Arenberg 31, 3001 Leuven, Belgium

**Supplementary figure 1**

**

**

**Supplementary figure 1:** Rockwool EC after 7 days of control (white), mild (EC of 5 dS/m; [NaCl] = 47 mM, light gray) and severe (EC of 10 dS/m; [NaCl] = 94 mM, dark grey) salt stress.

**Supplementary table 1:** RT-qPCR NCED primers.

|  | **FW 5’-3’** | **RV 5’-3’** |
| --- | --- | --- |
| **NCED1** | AGGCAACTGTGAAGCTTCCATCAAG | TCCATTAAAGAGGAATATTCCGGGGAC |
| **NCED2** | TGGTTTTCATGGGACATTCATTAGC | ATCTCCCTTCTCAACTCCCTATTCC |
| **NCED6** | GTGGGTCACATTCCGTCTAAT | TCATACCGTCACCGTCAAATAA |
